# Clomiphene Citrate Treatment as an Alternative Therapeutic Approach for Male Hypogonadism: Mechanisms and Clinical Implications

**DOI:** 10.3390/ph17091233

**Published:** 2024-09-18

**Authors:** Yao-Cheng Wu, Wen-Wei Sung

**Affiliations:** 1 School of Medicine, Chung Shan Medical University, Taichung 40201, Taiwan; s0801086@gm.csmu.edu.tw; 2 Department of Urology, Chung Shan Medical University Hospital, Taichung 40201, Taiwan; 3 Institute of Medicine, Chung Shan Medical University, Taichung 40201, Taiwan

**Keywords:** clomiphene, hypogonadism, testosterone, replacement therapy, sexual function, infertility

## Abstract

Male hypogonadism, which is characterized by low testosterone levels, has a significant impact on male sexual function, overall health, and fertility. Testosterone replacement therapy (TRT) is the conventional treatment for this condition, but it has potential adverse effects and is not suitable for men seeking to conceive. Testosterone plays an essential role in male sexual function, metabolism, mood, and overall well-being. Clomiphene citrate, a drug originally developed for female infertility, has recently gained attention as an off-label treatment for male hypogonadism. By blocking the negative feedback of estrogen on the hypothalamus and pituitary glands, clomiphene stimulates gonadotropin secretion, leading to increased endogenous testosterone production, which, in turn, improves sperm parameters and fertility and alleviates the symptoms of hypogonadism. Regarding the safety profile of clomiphene compared with TRT, clomiphene appears to confer a lower risk than TRT, which is associated with adverse effects such as polycythemia. Furthermore, combination therapy with clomiphene and anastrozole or human chorionic gonadotropin has been investigated as a potential approach to enhancing the effectiveness of treatments for improving hypogonadism symptoms. In conclusion, clomiphene citrate may offer a promising alternative to TRT for men with hypogonadism, particularly those desiring fertility preservations. However, its long-term efficacy and safety remain inadequately understood. Future research should focus on exploring the benefits of combination therapies and personalized treatment strategies based on individual patient characteristics.

## 1. Introduction

Male hypogonadism, a testosterone deficiency, is defined as a total testosterone level < 300 ng/dL according to the American Urology Association [1]. Testosterone, a major androgen in men, is a crucial hormone for development, sexual function, and organ function [2]. A decline in testosterone levels is associated with sexual symptoms, depression, fatigue, and impaired spermatogenesis [3,4]. In the United States, the estimated number of newly diagnosed cases of androgen deficiency annually among men between 40 and 69 years of age is 481,000, according to the Massachusetts Male Aging Study [5]. Male hypogonadism causes a deterioration in testicle function and androgen production and is primarily attributed to central obesity, diabetes, and poor health conditions. Additionally, symptomatic androgen deficiency is exacerbated with age [6,7]. The prevalence of hypogonadism is significantly higher in men aged 70–79 years (18.4%) than in men aged <70 years (3.1%–7.0%) [8]. Furthermore, low testosterone levels have been reported to correlate with several comorbidities, including hypertension, coronary heart disease, peripheral arterial disease, stroke, obesity, and diabetes mellitus (DM) [9,10,11]. Especially in patients with type 2 DM (T2DM), a pronounced incidence of secondary hypogonadism has also been reported [12].

Testosterone replacement therapy (TRT) is the most common therapeutic approach for hypogonadism in men. Regarding sexual dysfunction, TRT improved sexual activity, sexual desire, and erectile function in men with low testosterone levels [13,14]. In addition, it improved skeletal muscle mass, bone mineral density, mood, and quality of life [15,16,17]. However, it is not suitable for men seeking to conceive, and its side effects can be serious. Exogenous testosterone use inhibits gonadotropin secretion because of negative feedback, which inhibits intratesticular testosterone levels and overall testosterone production. This may suppress sperm production and lead to infertility [18,19]. Furthermore, TRT is associated with cardiovascular risk, polycythemia, erythrocytosis, prostate-related events, lipid alterations, gynecomastia, and sleep apnea, among others [16,20,21,22,23,24].

Mounting evidence has revealed effective alternative treatments for male hypogonadism, including aromatase inhibitors (AIs) and selective estrogen receptor modulators (SERMs). AIs such as letrozole and anastrozole can suppress the conversion of testosterone into estradiol and inhibit negative feedback on the hypothalamic–pituitary–gonadal (HPG) axis. Since the pituitary gland primarily senses testosterone levels through estrogen concentrations, inhibiting estrogen leads to an increase in luteinizing hormone (LH) production, which in turn enhances endogenous testosterone secretion and sperm production [25]. Tamoxifen is a SERM that has been used to treat male infertility thanks to its ability to inhibit the negative feedback of estrogen on the hypothalamus and pituitary glands. This results in increased follicle-stimulating hormone (FSH) and testosterone levels, thereby improving sperm parameters [26]. However, AIs and SERMs are not approved for the treatment of male hypogonadism and are used as off-label treatments.

Clomiphene citrate, a SERM originally developed for female infertility, is an alternative therapeutic option for male hypogonadism. Clomiphene competitively inhibits 17β-estradiol and blocks estrogen receptors in the hypothalamic arcuate nucleus [27]. This action disrupts the negative feedback of estrogen at the hypothalamus and pituitary gland and stimulates the production of gonadotropin, resulting in endogenous testosterone secretion [28,29]. Unlike TRT, clomiphene can maintain high FSH and LH levels, as well as intratesticular testosterone levels, thereby improving spermatogenesis and increasing sperm concentration and motility [30,31]. Moreover, the advantages of the long-term use of clomiphene over that of TRT are its low cost, high safety profile, and minor side effects [32,33]. The aim of the present literature review is to investigate the pharmacological mechanisms and potential beneficial effects of clomiphene citrate administration as a therapeutic approach for male hypogonadism.

## 2. HPG Axis in Men

**Figure 1:**
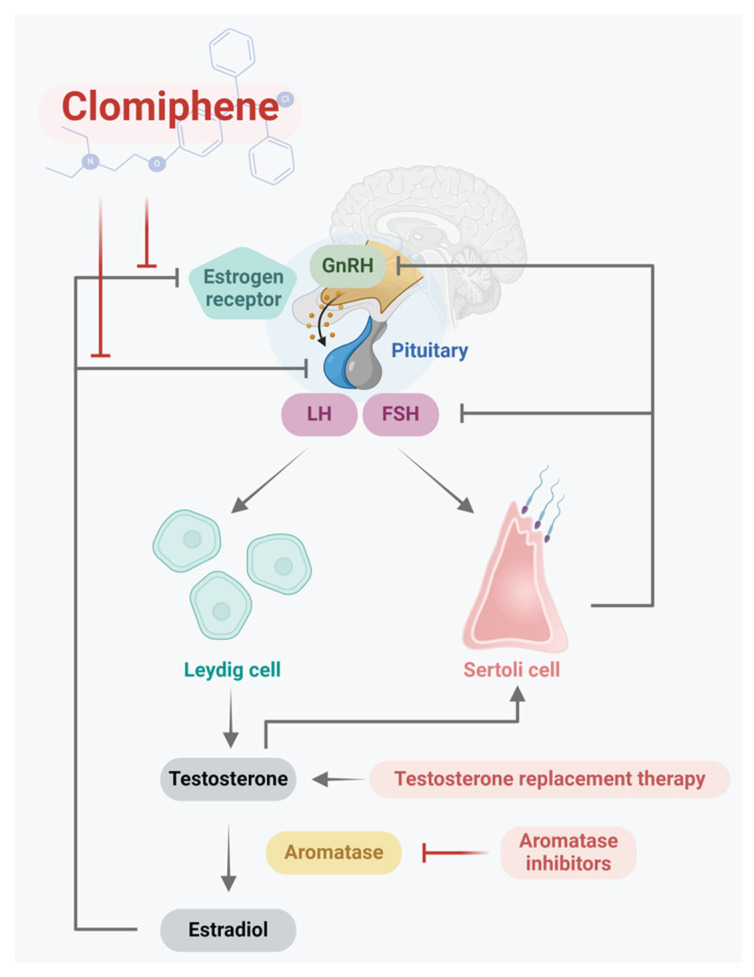
Illustration of the targets of clomiphene citrate in the HPG axis in males. The hypothalamus secretes GnRH, which is delivered to the pituitary gland to stimulate the biosynthesis of the gonadotropins LH and FSH. LH functions within Leydig cells and results in the secretion of testosterone. Testosterone can be modified via aromatase for estradiol and acts as a negative feedback signal in the HPG axis. Clomiphene citrate can reduce this signal and weaken the negative feedback loop to improve testosterone synthesis.

Reproductive functions in both sexes are regulated by the coordination of the HPG axis in a cascading effect. The HPG axis is composed of the hypothalamus, pituitary gland, and testes in males. Physiologically, the hypothalamus secretes gonadotropin-releasing hormone (GnRH) in a pulsatile pattern. GnRH is delivered to the pituitary gland and stimulates the biosynthesis of the gonadotropins LH and FSH (Figure 1). LH functions within Leydig cells and increases the secretion of androgens, including testosterone. Testosterone plays an important role in male sex differentiation, biological functions, and sexual function [7]. FSH acts on Sertoli cells, promoting the synthesis of androgen-binding proteins and aromatase enzymes [34], and sustains the microenvironment for spermatogenesis [35].

## 3. Testosterone Synthesis

LH is secreted in a pulsatile pattern from the pituitary gland into the circulation, attaching to receptors on the Leydig cell plasma membrane [36]. The binding of LH to an LH receptor (LHR) results in the coupling of LHR to G proteins, initiating a cascade of reactions in the cell that involves the biosynthesis of adenosine 3′,5′-cyclic monophosphate (cAMP) and the activation of protein kinase A [37]. Elevated cAMP levels facilitate the transfer of cholesterol to the inner membrane of the mitochondria. Cholesterol is converted into pregnenolone via side-chain cleavage by the cytochrome P450 enzyme CYP11A1. Subsequently, pregnenolone exits the mitochondria, enters the smooth endoplasmic reticulum, and is metabolized into progesterone and androstenedione by 3β-hydroxysteroid dehydrogenase and cytochrome P450 C17 hydroxylase/17,20-lyase [38]. Eventually, type 3 17β-hydroxysteroid dehydrogenase converts androstenedione into testosterone, which is then released into the bloodstream [39]. Other androgens such as dehydroepiandrosterone (DHEA) are also produced in the testes and added into the circulation. However, less testosterone is derived from the DHEA in the bloodstream, which contributes to limited daily testosterone production in men [40].

## 4. Biological Function of Testosterone

Testosterone plays a crucial role in early male sex differentiation; reproductive organ development; and biological functions, including metabolism, mood, cognition, muscle strength, bone density, and cardiovascular function.

As the primary male hormone, testosterone regulates sexual functions such as libido, fertility, and spermatogenesis. Spermatogenesis occurs in the seminiferous tubule with the help of Sertoli cells, which can secrete glycoproteins that promote the progression of spermatogonia to spermatozoa [41,42]. Sertoli cells also have testosterone and FSH receptors, which collectively aid in sperm maturation [43] and prevent germ cell apoptosis [44,45]. In addition, testosterone is important not only in maintaining the physiological process of penile erection but also in stimulating sexual desire [46]. Therefore, low testosterone levels lead to the absence of erection or penetration impairment, which hinders fertility [47].

As testosterone is associated with fatty acid metabolism, glucose regulation, and lipid deposition, low testosterone levels are an independent risk factor of metabolic syndrome [11,48] and can cause elevated fat mass, increased visceral adiposity, decreased insulin sensitivity, and abdominal or central obesity [49,50]. In addition, increased adiposity elevates leptin levels and directly inhibits testosterone production in Leydig cells [51]. Studies have reported low testosterone levels in men with obesity, as well as their direct proportionality to the degree of obesity [52].

Low testosterone levels are common in patients with T2DM [12]. Research has shown that at least 25% of men diagnosed with T2DM exhibit testosterone deficiency [53]. However, the possible pathophysiological mechanism remains undefined, although various mechanisms have been investigated. Chronic inflammation, including elevated tumor necrosis factor-α and interleukin-1β expression levels, is a mechanism that could contribute to the inhibition of hypothalamic GnRH and LH secretions and the development of hypogonadism in patients with T2DM [54,55]. In a previous study, low testosterone levels also increased the concentrations of inflammatory mediators such as C-reactive protein, which is markedly elevated in patients with T2DM [56]. Low testosterone levels could reduce insulin sensitivity [53]. In men with hypogonadotropic hypogonadism and T2DM, insulin sensitivity increases after exogenous testosterone administration [57].

Furthermore, low testosterone levels have been demonstrated to have an impact on mood and psychological health. Endogenous testosterone levels and the external administration of the sex steroid can lead to significant mood and behavioral alterations. In men with hypogonadism, exogenous testosterone administration improved positive mood parameters and decreased negative mood parameters, as measured using a Likert rating scale, as well as patient quality of life [17,58].

## 5. Male Hypogonadism

According to the original disorders, male hypogonadism can be classified as either primary or secondary. Distinct causes and clinical symptoms have been found in these two categories. Primary hypogonadism (hypergonadotropic hypogonadism) originates from testicular dysfunction, causing a decrease in testosterone levels and impairment of spermatogenesis [59]. Low testosterone levels lead to a compensatory increase in gonadotropin secretion, which leads to high/normal serum LH and FSH levels [60]. Common causes of primary hypogonadism include Klinefelter’s syndrome, medications, malignancies, and aging [61]. On the contrary, secondary hypogonadism (hypogonadotropic hypogonadism) results from the testes not being adequately stimulated by gonadotropins and is marked by reduced testosterone levels, normal/reduced gonadotropin levels, and reduced spermatogenesis [62]. Its common causes include pituitary disorders, hypothalamic dysfunction, hyperprolactinemia, medications, obesity, T2DM, and aging [63]. Both primary and secondary hypogonadism can have adverse effects on sperm concentrations or spermatogenesis.

## 6. Male Infertility

Infertility is defined as the inability to achieve a successful pregnancy after 12 months or more of appropriate unprotected intercourse [64]. According to estimates, 7% of men are infertile worldwide [65]. Male infertility is due to abnormal sperm parameters resulting from three aspects related to this pathological condition: secondary hypogonadism, seminal outflow obstruction, and testicular dysfunction (e.g., primary hypogonadism) [66,67]. Spermatozoa are produced in seminiferous tubules. The HPG axis-related hormones, namely GnRH, LH, FSH, testosterone, and inhibin, are involved in sperm production. In spermatogenesis, FSH and testosterone are necessary for sperm maturation [43]. Normally, spermatozoa are stored in the cauda epididymis. During ejaculation, spermatozoa are transported from the epididymis to the urethral meatus. Disruptions of any of these steps may lead to male infertility.

## 7. Treatment Options for Male Hypogonadism

The primary treatment for symptomatic hypogonadism (total testosterone < 12 nmol/L) in men is TRT (Table 1), which is suitable for patients without contraindications [6]. Research has demonstrated that TRT exerts a positive effect on sexual dysfunction. Sexual activity and desire improved significantly with TRT in men with low testosterone levels [13,68]. Corona et al. [14] also showed that TRT improved erectile function in hypogonadal men, as measured using the International Index of Erectile Function (IIEF). Furthermore, physical and psychological changes were observed after TRT administration. TRT can improve skeletal muscle mass, grip strength, and bone mineral density and can reduce adipose tissue mass [16]. For psychological functions, studies have shown that TRT can improve mood and quality of life in men with hypogonadism [17,58,69]. A decrease in mortality was also observed in a retrospective observational cohort study [70].

Although TRT is an effective therapeutic approach to resolving the symptoms of hypogonadism, the side effects of TRT can be serious. Cardiovascular-related events are the main adverse events that occur after exogenous testosterone administration, for which the Food and Drug Administration (FDA) issued an advisory warning in 2015. Two observational studies [22,23] demonstrated that TRT increased cardiovascular risk, which was confirmed by a meta-analysis of randomized trials [21]. In people with hypogonadism who had undergone coronary angiography, TRT increased the risk of all-cause mortality, ischemic stroke, and myocardial infarction [22]. Furthermore, exogenous testosterone administration has been shown to cause negative feedback on the HPG axis, inhibiting gonadotropin secretion. As LH and FSH levels decrease, intratesticular testosterone levels and overall testosterone production are also reduced, leading to impaired spermatogenesis [18]. Thus, TRT is not suitable for men seeking to conceive. TRT has also been found to be related to other adverse events, including polycythemia, erythrocytosis, prostate-related events, lipid alterations, gynecomastia, and sleep apnea [16,20].

Non-hormonal treatment strategies for male hypogonadism have emerged as viable therapeutic options, including AIs and SERMs. AIs, particularly letrozole and anastrozole, can suppress the conversion of testosterone into estradiol and inhibit negative feedback on the HPG axis [25,71]. This increases endogenous testosterone and FSH production, thereby improving sperm production and parameters. However, compared with TRT and the placebo, the reduction in estrogen caused by AIs has been associated with lower bone mineral density in men with low testosterone levels [72]. Therefore, further randomized controlled trials (RCTs) are needed to validate the long-term efficacy and safety of AIs in treating hypogonadism in men.

Another non-hormonal therapeutic option is the use of tamoxifen, a SERM. Tamoxifen is an estrogen agonist or antagonist depending on the tissue, disrupting the negative feedback of estrogen on the hypothalamus and pituitary gland [26]. The efficacy of tamoxifen in treating male infertility remains controversial. While tamoxifen has been shown to increase the levels of FSH, LH, estradiol, and testosterone, leading to improvements in sperm density and the number of viable spermatozoa, RCTs have reported no significant improvements in sperm motility, sperm morphology, or certain ejaculate parameters [26,73,74,75]. Given the widespread off-label use of tamoxifen for treating male hypogonadism and infertility, additional supporting data and further research are needed to clarify its efficacy.

## 8. Clomiphene

In the 1960s, clomiphene citrate (2-[4-[(Z)-2-chloro-1,2-diphenylethenyl]phenoxy]-*N*,*N*-diethylethanamine) first brought about a revolutionary change in the treatment of female infertility and polycystic ovarian syndrome thanks to its ability to induce ovulation. Recently, clomiphene has been used for the off-label treatment of male hypogonadism despite not being approved by the FDA, being the most commonly prescribed medication by urologists for the empirical medical therapy of male infertility according to a cohort study of the American Urological Association [76]. Clomiphene is a racemic mixture composed of 38% zuclomiphene (*cis*-isomer) and 62% enclomiphene (trans-isomer), which is excreted through the intestines and has a half-life of 5 days [27]. Structurally, clomiphene is a derivative of triphenylethylene, substituted with a chloride anion and an aminoalkoxy side chain, and is related to another nonsteroidal SERM, tamoxifen [77]. It is also classified as a catechol-estrogen, which is attributed to the presence of a diethylamino group [27].

## 9. Hormonal Measurement

As a SERM, clomiphene exhibits both estrogenic and antiestrogenic properties, based on its target tissue [78]. As an anti-estrogen, clomiphene competitively inhibits 17β-estradiol and blocks estrogen receptors in the hypothalamic arcuate nucleus [27]. This inhibition disrupts the negative feedback of estrogen at both the hypothalamus and pituitary gland, leading to an increase in GnRH secretion, which subsequently elevates FSH and LH levels, potentially inducing ovarian follicular growth. However, clomiphene also results in endometrial growth, which may have an adverse impact on implantation [79]. The current use of clomiphene includes the treatment of male hypogonadism. Studies have demonstrated that clomiphene could cause significant increases in testosterone, LH, and FSH levels and testosterone/estradiol ratios, thereby positively influencing sperm concentration and motility [30,31,80].

Ramasamy et al. [81] showed similar elevations in serum testosterone levels in men with hypogonadism treated with clomiphene compared with those treated with testosterone gels (504 and 412 ng/dL; *p* = 0.31). However, their testosterone levels were lower than those achieved with testosterone injections (1014 ng/dL; *p* < 0.01). Compared with aromatase inhibitors such as anastrozole, clomiphene led to significantly elevated testosterone levels (408 and 571 ng/dL; *p* = 0.04) [82]. Of note, estradiol levels decreased in the anastrozole group while they increased in the clomiphene group. This difference may raise concerns about the potential decrease in bone mineral density associated with anastrozole use. No direct comparisons of testosterone concentrations between treatments with clomiphene and tamoxifen were found.

In addition to testosterone, LH and FSH are important for reproductive function. LH stimulates Leydig cells for testosterone production, while FSH acts on Sertoli cells in the seminiferous tubules to support spermatogenesis. Many studies have observed increases in LH and FSH levels in men with hypogonadism treated with clomiphene [33,83,84]. By contrast, TRT inhibits LH and FSH production as a result of negative feedback, leading to intratesticular testosterone depletion [85,86]. The effects of aromatase inhibitors, such as testolactone, anastrozole, and letrozole, on LH and FSH levels remain a topic of debate [82,87,88,89,90,91].

## 10. Treatment of Hypogonadism Symptoms

The severity of androgen deficiency in men treated with clomiphene has been evaluated using the Androgen Deficiency in Aging Males (ADAM) questionnaire and quantitative ADAM (qADAM) score. The ADAM questionnaire consists of 10 yes/no questions associated with libido, erection strength, muscular strength, energy, and other hypogonadism symptoms, having 97% sensitivity and 30% specificity [92,93]. A more recent questionnaire, qADAM, has since been developed, substituting the binary yes/no answers with a scale of 1 to 5 (1 indicating maximum severity of a symptom and 5 indicating the absence of a given symptom) [94]. qADAM was found to be significantly correlated with serum testosterone level, the Sexual Health Inventory for Men (SHIM) score, and the Expanded Prostate Cancer Index Composite hormonal/sexual score.

Many cohort studies have observed an improvement in ADAM or qADAM scores in men treated with clomiphene. Taylor et al. [33] compared the efficacy of testosterone gel replacement therapy with that of clomiphene therapy (commenced with 50 mg orally every other day, titrated to 25–100 mg every other day to achieve a serum testosterone level in the middle–normal range) in men with hypogonadism. The ADAM score improved after treatment (from 4.9 pre-treatment to 2.1 post-treatment, *p* < 0.05), with a mean decline in the score of 2.8. Katz et al. [95] prospectively evaluated the efficacy of clomiphene in patients with a mean age of 29 years (22–37 years, SD = 3). After receiving 25 mg of clomiphene every other day (titrated to 50 mg every other day to achieve a testosterone level of 550 ± 50 ng/dL) for at least 6 months, the patients’ ADAM scores decreased from the baseline in all variables except height loss and decreased significantly in five variables. However, 10% of the population did not experience improvement. Chandrapal et al. [96] revealed an improvement in ADAM score after the administration of 50 mg of clomiphene every day or every other day for 12 months. Moskovic et al. [97] also observed a significant improvement in ADAM score after long-term use of clomiphene.

A randomized study [98] demonstrated that clomiphene, hCG, and clomiphene + hCG therapies improved qADAM scores after 3 months. However, no randomized study has reported improvements in ADAM score; neither a randomized double-blind prospective study [82] in patients who received clomiphene or anastrozole after 12 weeks nor a randomized double-blind placebo-controlled study of 12 weeks of clomiphene or the placebo [99] observed significant differences in ADAM scores.

Only a few studies have investigated the effectiveness of clomiphene therapy for the treatment of sexual and erectile dysfunctions. In 1995, Guay et al. [100] conducted a double-blind study with clomiphene administration in 17 patients with secondary hypogonadism. In their study, no significant differences were found in nocturnal penile tumescence and rigidity test scores. Later, Guay et al. [101] revealed that free testosterone levels improved in all patients with erectile dysfunction (ED) and that >75.1% of patients showed improved sexual function after clomiphene therapy for 4 months. However, a cohort study showed no significant difference in SHIM score for ED symptoms after clomiphene treatment in men with hypogonadism [96]. Helo et al. [82] found that neither clomiphene nor anastrozole administration significantly improved the outcome in terms of IIEF and Erection Hardness Scale (EHS) scores.

Clomiphene treatment has its limitations. Unlike TRT, clomiphene targets the HPG axis, necessitating a functional HPG axis for its anti-estrogenic effects to be effective [102]. Additionally, clomiphene is not suitable for treating organic central hypogonadism [103], a condition that has often been inadequately distinguished in previous studies. The lack of high-quality RCTs and long-term follow-up data further raises concerns about the justification of clomiphene citrate as a treatment for sexual symptoms in cases of functional central hypogonadism.

## 11. Treatment of Infertility

Increases in LH and FSH levels by clomiphene administration result in a pronounced stimulation of spermatogenesis, promoting somatic cell function to support spermatogenesis [104]. Sperm parameters, sperm concentration, motility, and morphology have been demonstrated to improve in men with infertility due to hypogonadism treated with clomiphene therapy [83,105,106,107,108]. Huijben et al. [30] performed a meta-analysis that included both clinical trials and observational studies. They reported significant increases in sperm concentration during clomiphene treatment, with a mean increase of 8.38 × 10^6^/mL (95% confidence interval [CI], 5.17–11.59 × 10^6^/mL; *p* < 0.00001; *I*^2^ = 87%); a significant improvement in sperm motility, with a mean increase of 8.14% (95% CI, 3.83–12.45%; *p* < 0.00001; *I*^2^ = 76%); and slightly, though not statistically significantly, improved sperm morphology during clomiphene treatment (from 37.79% to 41.23%).

In 1992, the World Health Organization conducted a multicenter randomized double-blind study that included 190 infertile couples (normal females) to determine the fertility outcomes of clomiphene therapy [109]. Male patients received a daily dose of 25 mg of clomiphene or a placebo for 6 months. The cumulative life pregnancy rates at 8 months did not show significant differences between the placebo and clomiphene groups (11.7% vs. 8.1%). Although a meta-analysis indicated an improvement in pregnancy rate after treatment with 50 mg of clomiphene daily, this effect was not observed with a daily dose of 25 mg [110].

## 12. Safety

Clomiphene is typically viewed as a relatively tolerable medication in clinical practice. However, due to its dual action as both an estrogen agonist and antagonist, it is associated with a range of adverse effects. The anti-estrogenic properties of clomiphene can lead to side effects such as hot flashes, headaches, and visual disturbances [111,112]. Additionally, other reported adverse events include mood alterations, dizziness, gynecomastia, breast and nipple tenderness, and testicular enlargement [113].

A retrospective study [32] reviewed 400 patients treated with clomiphene over an 8-year period, with a specific focus on long-term outcomes, particularly those involving treatment durations of more than 3 years. That study found that 88% of patients who used clomiphene for over 3 years achieved eugonadal testosterone levels, while 77% experienced improvements in hypogonadal symptoms. The safety profile of clomiphene in that long-term cohort was favorable, with only 8% of patients reporting side effects, none of which were severe or led to long-term adverse events. The most commonly reported side effects included mood changes, blurred vision, and breast tenderness, which are consistent with the known side effects of clomiphene observed in shorter-term studies.

However, severe clomiphene-induced hypertriglyceridemia has been reported in two case studies [114,115]. In light of these reports, we investigated the impact of clomiphene therapy on lipid parameters. Da Ros et al. [116] observed lower total cholesterol levels in men with hypogonadism who were receiving 25 mg of clomiphene daily. However, the total cholesterol levels did not significantly change in other studies [33,99]. Previous studies have compared body mass index (BMI) before and during the course of clomiphene treatment. Among these studies, only one demonstrated a significant decrease in BMI after 3 years of clomiphene treatment [97], whereas the remainder showed no significant differences in BMI before and during treatment [98,99,117].

Elevated testosterone levels can lead to polycythemia. Men with polycythemia exhibit higher incidence rates of major adverse cardiovascular events, venous thromboembolic events, and stroke [118,119]. TRT has been shown to significantly raise hemoglobin counts and hematocrit levels, particularly in patients receiving intramuscular injections [118,120,121]. Therefore, secondary polycythemia is a main side effect of TRT, with the FDA warning against its use [24]. Although elevated serum testosterone levels can also be observed during clomiphene therapy, no information is available regarding the possibility of secondary polycythemia occurring in men receiving clomiphene therapy [97]. Wheeler et al. [20] indicated that the prevalence of polycythemia is significantly lower in men receiving clomiphene therapy than in those receiving TRT (1.7% vs. 11.2%).

In a case report, an opposite effect, azoospermia, was demonstrated in patients treated with clomiphene therapy. Pasqualotto et al. [122] also found azoospermia in patients with oligospermia after treatment with clomiphene therapy. Therefore, further research is required to confirm the side effects of clomiphene therapy on fertility function.

## 13. Population Variability in Treatment Response

Male obesity is believed to be one of the most prevalent causes of secondary hypogonadism. Adipose tissue exhibits high aromatase activity, which converts testosterone into estradiol, subsequently increasing serum estradiol levels and contributing to hypogonadism [123]. Clomiphene has been identified as an effective treatment option for male obesity-associated secondary hypogonadism (MOSH). One RCT demonstrated that clomiphene significantly increased the levels of total testosterone, free testosterone, LH, FSH, estradiol, and sex hormone-binding globulin (SHBG), as well as showing improvements in ADAM score [99]. Additionally, the clomiphene group showed significant increases in lean mass, fat-free mass, and muscle mass. A meta-analysis study further confirmed a significant increase in testosterone levels after receiving clomiphene in MOSH patients [124]. In terms of fertility, clomiphene also enhanced sperm concentration and motility in men with obesity [125]. However, the changes in pregnancy rate require further clarification.

The efficacy of TRT in treating male hypogonadism associated with obesity has been studied. While testosterone gel demonstrated a beneficial effect in increasing total testosterone levels, it also led to a decrease in LH and FSH levels, along with impaired spermatogenesis, in obese men [126]. AIs, such as anastrozole and letrozole, have also shown therapeutic effects in this population. A meta-analysis [127] showed that AIs significantly increased total testosterone and LH levels while decreasing estradiol levels. In addition, an RCT found that obese men in the weight loss plus anastrozole group had increased testosterone and decreased estradiol levels compared with the placebo group [128]. However, there are limited data on the resolution of hypogonadal symptoms and fertility outcomes in obese men treated with AIs. A retrospective chart review showed improvements in sperm concentration, total motile count, and strict morphology after anastrozole treatment [129], but further studies are needed to confirm these findings. There is also a lack of data on the effects of tamoxifen on hypogonadism in obese men.

Young men represent a unique population of concern when considering treatments for hypogonadism because of their heightened interest in preserving fertility. In young men with hypogonadism, with a mean age of 29 years, clomiphene treatment resulted in significant increases in total testosterone, free testosterone, LH, FSH, and estradiol levels. Regarding the ADAM questionnaire, 9 out of 10 questions showed improvements, with the exception of loss of height [95]. Similar hormonal effects were observed in post-pubertal males with hypogonadism and obesity, aged 18–21 years, with elevations of testosterone, LH, and FSH levels after clomiphene treatment [117]. Despite the benefits of restoring testosterone levels and alleviating hypogonadal symptoms [130], concerns about the potential for irreversible infertility with TRT make clomiphene a promising alternative for young men with hypogonadism who wish to preserve their fertility. AIs and tamoxifen also show beneficial effects in treating hypogonadism without impairing fertility; however, specific studies focusing on young hypogonadal men are lacking.

## 14. Combination Therapy

The efficacy of combining clomiphene with other treatments has been investigated in various studies. In a randomized study, Habous et al. [98] demonstrated that testosterone levels were significantly increased from baseline in men with hypogonadism treated with clomiphene, human chorionic gonadotropin (hCG), or clomiphene + hCG, but the differences were not statistically significant. Additionally, combining clomiphene with anastrozole effectively maintained therapeutic testosterone levels in patients with hyperestrogenemia or a low testosterone:estradiol ratio [131]. Dual therapy with clomiphene (50 mg daily) and intracavernosal alprostadil (10–20 μg daily) also resulted in significant increases in total testosterone, FSH, and LH levels [132].

Combination therapy with clomiphene has shown efficacy in improving hypogonadal symptoms and fertility. Clomiphene combined with alprostadil improved IIEF-15 subscores and EHS scores in men with late-onset hypogonadism and penile vasculogenic erectile dysfunction who were unresponsive to the phosphodiesterase type 5 inhibitor [132]. Furthermore, combining clomiphene (25 mg daily) with vitamin E (400 mg daily) led to higher sperm counts, improved progressive sperm motility, and a higher pregnancy rate (36.7% in the combination group compared with 13.3% in the placebo group; odds ratio 3.76; 95% CI 1.03–13.64) [133].

However, safety is a potential concern with combination therapy. Although the combination of clomiphene and anastrozole demonstrated a generally acceptable safety profile [131], 11 patients (21.5%) reported side effects, including anxiety or irritability (n = 5), decreased libido (n = 4), and elevated hematocrit levels (n = 2), leading to treatment discontinuation in four cases (8%). However, men with obesity may be more susceptible to developing hyperestrogenemia when on clomiphene monotherapy [131]. Therefore, the combination of clomiphene and anastrozole remains a promising therapeutic option, as it can effectively maintain therapeutic testosterone levels, provided that their hematocrit levels are closely monitored. The efficacy of other combination options or within different study populations requires further investigation and clarification.

## 15. Treatment Cost

Clomiphene is considered a cost-effective medication compared with TRT; however, extensive direct comparison research related to male hypogonadism is still lacking. Taylor et al. [33] estimated the monthly cost of clomiphene (50 mg orally every other day) compared with testosterone gel (5 gm daily) and showed that clomiphene was less expensive, with a monthly cost of USD 83 compared with USD 265 for testosterone gel.

However, no other direct comparisons between clomiphene and other testosterone formulations or non-hormonal treatments have been reported.

## 16. Conclusions

In conclusion, clomiphene effectively increases serum testosterone levels in men with hypogonadism by inhibiting the negative feedback of estrogen and increasing LH and FSH levels, as well as intratesticular testosterone levels, thereby improving sperm parameters. Furthermore, clomiphene administration showed a positive effect on fertility and hypogonadism symptoms without major adverse effects, such as polycythemia and infertility, which are commonly associated with TRT. Therefore, clomiphene is a promising alternative, particularly for patients aiming to preserve fertility. However, the long-term efficacy and safety profiles of clomiphene are not fully understood. Future research should focus on elucidating these aspects and exploring the benefits of combination therapies and personalized treatment strategies based on individual patient characteristics.

## Figures and Tables

**Table 1:** Comparison of therapeutic options for hypogonadism. Aromatase inhibitors, AI; Food and Drug Administration, FDA; gonadotropin-releasing hormone, GnRH; hypothalamic–pituitary–gonadal, HPG; randomized controlled trial, RCT; testosterone replacement therapy, TRT.

Therapy	Mechanism of Action	Side Effects	Benefits	Risks
Testosterone Replacement Therapy	Administers exogenous testosterone through various formulations (oral, intramuscular, transdermal, subdermal, buccal, and nasal).	Associated with potential risks, including cardiovascular events, impaired fertility, obstructive sleep apnea, and erythrocytosis.	Supported by substantial long-term RCT evidence; FDA-approved to treat men with hypogonadism.	Risk of significant adverse effects, particularly erythrocytosis and infertility.
Aromatase Inhibitors	Inhibit the enzymatic conversion of testosterone to estradiol, reducing negative feedback on the HPG axis.	Common side effects include nausea, headache, and hot flashes. Less frequent effects include libido changes and elevated liver enzymes. Decreased bone mineral density has been reported.	Improves hypogonadal symptoms and fertility outcomes without causing major side effects.	Potential risk of decreased estradiol interfering with bone metabolism and associated symptoms; lack of long-term RCT data.
Tamoxifen	Blocks estrogen’s negative feedback on the hypothalamus and pituitary gland, leading to increased GnRH production, which subsequently boosts endogenous testosterone secretion.	Potential adverse effects include weight gain, sexual dysfunction, hot flashes, and neurocognitive impairment; rare reports of infertility and idiopathic gynecomastia.	Improves hypogonadal symptoms and fertility outcomes without significant side effects or suppression of estradiol.	Requires a functional HPG axis; lacks robust RCT evidence.
Clomiphene		Reported side effects include headaches, visual disturbances, dizziness, gynecomastia, and testicular enlargement; rare instances of azoospermia have been documented.	Improves hypogonadal symptoms and fertility outcomes without significant side effects or suppression of estradiol; particularly promising for men with obesity-related hypogonadism or those prioritizing fertility preservation; cost-effective compared to TRT.	Requires a functional HPG axis; high-quality RCTs and long-term follow-up data are needed to fully establish its safety and efficacy.
